# Brain and bone metastasis from malignant thyroid carcinomas originating in western Saudi Arabia

**DOI:** 10.1186/1471-2164-15-S2-P64

**Published:** 2014-04-02

**Authors:** Ibtisam Baghallab, Manar Ata, Nafisa Abdullah Hassan, Shireen Hussain, Zuhoor Al-Mansouri, Deema Hussein, Jaudah Al-Maghrabi, Adeel Chaudhary, Mohammed Al-Qahtani, Hans-Juergen Schulten

**Affiliations:** 1 Center of Excellence in Genomic Medicine Research, King Abdulaziz University, Jeddah, Saudi Arabia; 2 Department of Pathology, Faculty of Medicine, King Abdulaziz University Hospital, Jeddah, Saudi Arabia; 3 Department of Pathology, King Faisal Specialist Hospital and Research Center, Jeddah, Saudi Arabia; 4 King Fahd Medical Research Centre, King Abdulaziz University, Jeddah, Saudi Arabia

## Background

We have previously investigated the frequency of gene mutations in RET and HRAS affecting medullary thyroid carcinomas (MTCs) and gene mutations in BRAF, HRAS, KRAS, and NRAS affecting non-medullary thyroid carcinomas (non-MTCs). Non-MTCs include follicular TCs (FTCs), anaplastic TCs (ATCs), and papillary TCs (PTCs) and its histological variants. In this study we surveyed the frequency of metastasis to the brain or bone in our case series of MTCs and non-MTCs.

## Materials and methods

We surveyed histopathological reports from two main hospitals in the Western region of Saudi Arabia for the presence of brain and/or bone metastasis in a case series of malignant TCs comprising 13 MTCs and 238 non-MTCs.

## Results

Our survey identified a small number of malignant TCs affected with bone and/or brain metastasis. Out of 115 PTCs, two cases were reported to have a metastasis to the bone and one case had a concurrent metastasis to the brain and to the skull bone. One case with a metastasis to the bone revealed in the mutational screening an uncommon K601E mutation in the BRAF gene whereas a rare insertion in BRAF codon 599 (insT599T) was identified in the case with the metastases to the brain and skull bone. This latter mutation was confined to the tumor. Out of 42 FVPTCs (follicular variants of PTCs), two cases were reported to have a metastasis to the bone. One of the FVPTCs displayed a HRAS Q61K mutation. In addition, one patient presenting with a micro PTC (tumor size ≤1 cm) had a brain metastasis originating from a breast carcinoma.

## Conclusions

Our survey reveals that especially, metastases to the brain are infrequent events in malignant TCs which is in concordance with other surveys [1]. The association of rare BRAF mutations with brain and bone metastasis from TC requires further investigations.

*This study was supported by King Abdulaziz City for Science and Technology* (*KACST*) *grant TK-0961-12.*
